# Supplementary material for: Identification of ligand-specific G protein-coupled receptor states and prediction of downstream efficacy via data-driven modeling
Source: eLife. 2021 Jan 28;10:e60715. doi: 10.7554/eLife.60715 (PMC7886328; doi:10.7554/eLife.60715)
Supplement: Transparent reporting form [file elife-60715-transrepform.docx]

***eLife’s* transparent reporting form**

We encourage authors to provide detailed information *within their submission* to facilitate the interpretation and replication of experiments. Authors can upload supporting documentation to indicate the use of appropriate reporting guidelines for health-related research (see [EQUATOR Network](http://www.equator-network.org/%20)), life science research (see the [BioSharing Information Resource](https://biosharing.org/)), or the [ARRIVE guidelines](http://www.plosbiology.org/article/info:doi/10.1371/journal.pbio.1000412) for reporting work involving animal research. Where applicable, authors should refer to any relevant reporting standards documents in this form.

If you have any questions, please consult our Journal Policies and/or contact us: [editorial@elifesciences.org](mailto:editorial@elifesciences.org).

**Sample-size estimation**

- You should state whether an appropriate sample size was computed when the study was being designed
- You should state the statistical method of sample size computation and any required assumptions
- If no explicit power analysis was used, you should describe how you decided what sample (replicate) size (number) to use

Please outline where this information can be found within the submission (e.g., sections or figure legends), or explain why this information doesn’t apply to your submission:

Simulation sample size can be found in the Methods section and in Table 2.

Convergence assessment can be found in Figure 2 (supplement 1-8), and Figure 3 (supplement 2-3).

**Replicates**

- You should report how often each experiment was performed
- You should include a definition of biological versus technical replication
- The data obtained should be provided and sufficient information should be provided to indicate the number of independent biological and/or technical replicates
- If you encountered any outliers, you should describe how these were handled
- Criteria for exclusion/inclusion of data should be clearly stated
- High-throughput sequence data should be uploaded before submission, with a private link for reviewers provided (these are available from both GEO and ArrayExpress)

Please outline where this information can be found within the submission (e.g., sections or figure legends), or explain why this information doesn’t apply to your submission:

We used iterative simulations methods with multiple replicas which converge after a number of iterations (Figure 2, supplement 1-6). The convergence of the free energy landscapes was further assessed with chain Markov Chain Monte Carlo bootstrapping (Figure 2, supplement 7). One simulation was also replicated from a different starting condition (Figure 2, supplement 9) to assess the dependence of initial conditions.

For the other simulations we assessed convergence by monitoring the drift of a high dimensional set of Collective Variables (Figure 3, supplement 2), and stratified cross validation between simulation replicas (Figure 3, supplement 3).

**Statistical reporting**

- Statistical analysis methods should be described and justified
- Raw data should be presented in figures whenever informative to do so (typically when N per group is less than 10)
- For each experiment, you should identify the statistical tests used, exact values of N, definitions of center, methods of multiple test correction, and dispersion and precision measures (e.g., mean, median, SD, SEM, confidence intervals; and, for the major substantive results, a measure of effect size (e.g., Pearson's r, Cohen's d)
- Report exact p-values wherever possible alongside the summary statistics and 95% confidence intervals. These should be reported for all key questions and not only when the p-value is less than 0.05.

Please outline where this information can be found within the submission (e.g., sections or figure legends), or explain why this information doesn’t apply to your submission:

In the first submission we provided p-values for the correlation between microswitch expectation values to downstream efficacy. The reviewers pointed out, for good reasons, that the correlation coefficient (i.e. the R-value) was better suited for this purpose. We have thus removed the use of the p-value. Details of the corresponding experimental values, including information on how their statistical properties were computed, are available in the referenced paper (van der Westhuizen, E. T., Breton, B., Christopoulos, A., and Bouvier, M. (2014))

The convergence of the free energy landscapes was further assessed with chain Markov Chain Monte Carlo bootstrapping (Figure 2, supplement 7). With this approach, we obtain a distribution of free energy landscapes from which we can compute statistical properties such as the standard deviation and identify outliers.

In Figure 3, supplement 2, we assessed convergence by monitoring the drift of a high dimensional set of Collective Variables. Here, the shaded area shows the upper and lower values of the drift by taking the standard deviation of the simulation replicas into account.

(For large datasets, or papers with a very large number of statistical tests, you may upload a single table file with tests, Ns, etc., with reference to sections in the manuscript.)

**Group allocation**

- Indicate how samples were allocated into experimental groups (in the case of clinical studies, please specify allocation to treatment method); if randomization was used, please also state if restricted randomization was applied
- Indicate if masking was used during group allocation, data collection and/or data analysis

Please outline where this information can be found within the submission (e.g., sections or figure legends), or explain why this information doesn’t apply to your submission:

N/A

**Additional data files (“source data”)**

- We encourage you to upload relevant additional data files, such as numerical data that are represented as a graph in a figure, or as a summary table
- Where provided, these should be in the most useful format, and they can be uploaded as “Source data” files linked to a main figure or table
- Include model definition files including the full list of parameters used
- Include code used for data analysis (e.g., R, MatLab)
- Avoid stating that data files are “available upon request”

Please indicate the figures or tables for which source data files have been provided:

All data used to reproduce the findings in this paper were uploaded to OSF, the codes are available on the lab’s GitHub repository.
